# Physiological Responses of the Bivalves *Mytilus galloprovincialis* and *Ruditapes decussatus* Following Exposure to Phenanthrene: Toxicokinetics, Dynamics and Biomarkers Study

**DOI:** 10.3390/ani13010151

**Published:** 2022-12-30

**Authors:** Mohamed Dellali, Khadija Mardassi, Abdel Halim Harrath, Lamjed Mansour, Octavian Pacioglu, Waleed Aldahmash, Saber Nahdi, Riadh Badraoui, Abdulwahed Fahad Alrefaei, Fehmi Boufahja

**Affiliations:** 1 Coastal Ecology and Ecotoxicology Unit, LR01ES14 Laboratory of Environment Biomonitoring, Faculty of Sciences of Bizerte, University of Carthage, Tunis 7021, Tunisia; 2 Zoology Department, College of Science, King Saud University, P.O. Box 2455, Riyadh 11451, Saudi Arabia; 3 National Institute of Research and Development for Biological Sciences, 060031 Bucharest, Romania; 4 Department of Biology, Laboratory of General Biology, University of Ha’il, P.O. Box 2440, Ha’il 81451, Saudi Arabia; 5 Section of Histology-Cytology, Medicine Faculty of Tunis, University of Tunis El Manar, La Rabta 1007, Road Djebal Lakhdhar, Tunis 1007, Tunisia; 6 Biology Department, College of Science, Imam Mohammad Ibn Saud Islamic University (IMSIU), P.O. Box 90950, Riyadh 11623, Saudi Arabia

**Keywords:** polycyclic aromatic hydrocarbons, chronic toxicity, bivalves, *stress on stress*, biomarkers, in silico modelling

## Abstract

**Simple Summary:**

The multiple impacts of polycylic aromatic hydrocarbon on the aquatic invertebrates were rarely assessed in a chronic way and multiple-species experiments, despite the clear advantage of better mimicking natural conditions compared to traditional acute and single-species-focused toxicological experiments. The application of such an approach is essential to lower the health risks for populations that regularly consume seafood. The data presented herein supported the use of *Mytilus galloprovincyalis* and *Ruditapes decussatus* as bioindicators of phenanthrene in water and/or sediment and proved the efficacy of the biomarkers’ assessment and molecular modelling in determining environmental thresholds and policies for governments.

**Abstract:**

The aim of the current study was to assess the multifaceted effects of the polycylic aromatic hydrocarbon phenanthrene, mainly used in the colouring, explosive, and pharmaceutical industries, on the physiology of two bivalve species with economic value as seafood, namely, the Mediterranean mussel *Mytilus galloprovincyalis* and the European clam *Ruditapes decussatus*. The current study assessed how the phenanthrene affected several biomarkers and biometric endpoints in both bivalves, based on an in vivo experiment in silico approach. The bivalves were exposed during four time slots (i.e., 7, 15, 21, and 28 days) to two concentrations of phenanthrene in water (50 µg/L and 100 µg/L). For the clam *R. decussatus*, an additional contamination of sediment was applied due their typical benthic lifestyle (50 µg/kg and 100 µg/kg). The phenanthrene significantly reduced the ability of bivalves to tolerate desiccation and their Median Lethal Time, and also inhibited the activity of the enzyme acetylcholinesterase in a time-dependent manner. The activity of catalase indicated that bivalves also experienced oxidative stress during the first 21 days of the experiment. The significant decline in catalase activity observed during the last week of the experiment for the mussel *M. galloprovincyalis* supported a depletion of enzymes caused by the phenanthrene. The phenanthrene has also toxicokinetic and toxicodynamic properties, as assessed by the in silico approach. Overall, the results obtained suggest that the bivalves *Ruditapes decussatus* and *M. galloprovincyalis* can be used as a sentinel species in monitoring studies to assess the environmental impact of phenanthene in marine ecosystems. The significance of our findings is based on the fact that in ecotoxicology, little is known about the chronic effects, the simultaneous use of multiple species as bioindicators, and the interactions molecular modelling.

## 1. Introduction

Marine ecosystems are increasingly polluted by a wide variety of substances with deleterious effects for fauna, including humans [1]. Polycyclic Aromatic Hydrocarbons (hereafter PAHs) are considered among the most toxic marine organic pollutants [2]. More than half of the PAHs that reach the marine habitats have their origins in human activities, such as civil combustion and industrial sources, maritime traffic, and petroleum accidents (6%) [3], as well as from the discharge of oil hydrocarbons in coastal waters [3]. According to the American list of priority pollutants [4], phenanthrene (three cycles), widely used in the colouring, explosive, and pharmaceutical industries [5], is of high concern given its high concentrations and persistence in marine habitats and bioaccumulation potential in marine fauna, increasing its toxicity. Phenanthrene is partially degradable in aquatic habitats; in just four weeks, up to 54% of the initial phenanthrene can be naturally degraded (OECDE301C method) [6], and its half-lives range from 64 to 800 days [7]. The degradation of phenanthrene appears to be slower in marine habitats compared to fresh waters because of its higher resistance to native bacteria [8]. Therefore, phenanthrene is considered a stable compound, with a high rate of bioaccumulation in consumers, and an affinity for substrates given its increased octanol/water partition coefficient (log *K_ow_* = 4.53) [9,10]. This PAH was proved to induce both chronic and acute toxic effects on wildlife, including marine biota [11,12].The toxicity of this PAH was previously investigated by experiments carried on specific sentinel species covering all trophic levels in marine food webs, such as the microalgae *Chlorella salina* (1–3 mg/L, 96 h, [13]), the copepod *Schizopera knabeni* (7.24 µmol/L, 96 h, [14]), the polychaete worm *Nereis (Neanthes) arenaceodentata* (100–1000 µg/L, 14 days, [15]), and the medaka *Oryzias melastigma* (100–800 µg/L, 25 days, [16]). In humans, the extent to which the phenanthrene is absorbed through the skin is less known, but traces of this substance were found in blood two days after its application on skin [17].

Similar to other marine species, the phenanthrene is suspected to induce detrimental effects on bivalves as well. Bivalves are particularly prone to bioaccumulation compared to other organisms, given their filter-feeding habit which leads to high concentrations accumulated in their tissues, with far-reaching hazardous effects on humans, given their consumption on large scale as seafood [18]. For these reasons the bivalves are considered efficient ecological indicators for the detection of early signs of stress induced by emerging pollutants [19,20]. Their global distribution, sedentary lifestyle, and low acquisition costs make the clams and mussels routinely used species in ecotoxicologic experiments [21,22,23,24].

Traditionally, the ecotoxicological studies relating pollutants and bivalves focused on acute (i.e., effects of short-term exposure, 24–96 h) and single-bivalve species. These classic studies also used a predefined set of endpoints, such as accumulation rate, shell allometry, mortality rate, and enzymatic activities. However, it is known that exposure to PAHs has multiple, often interlinking, effects on marine species and a comprehensive understanding of the overall process is still missing [19,20,21,22,23]. Further, the effects of chronic (i.e., longer-term) exposure are even less understood [25]. Therefore, the aim of the current study was to provide a better understanding of the multiple effects of phenanthrene on marine fauna by using three novel approaches. First, we simultaneously exposed two species of bivalves, the mussel *Mytilus galloprovincialis* and the clam *Ruditapes decussatus,* to increasing concentrations of phenanthrene. The second approach was to assess the toxic impact on both species by measuring the changes in time of various morphometric and mass end-points, as well as through neurotoxic and oxidative stress biomarkers, during a chronic exposure experiment (i.e., 7, 15, 21, and 28 days). In the third approach, bioaccumulation and high toxicity were linked to the toxicokinetics and toxicodynamics of phenanthrene, as well as its bioavailability. Such attributes mainly depend on the physico-chemical parameters of the pollutant itself, similar to several toxicants including PAHs using combined in silico and *in vivo*/*in vitro* approaches. Overall, the novelty and strength of the current study is twofold: it focused on the chronic rather than acute exposure and combined in vivo experiments with in silico tools, which is a relatively new investigation pathway in current ecotoxicological studies.

## 2. Material and Methods

**Figure 1:**
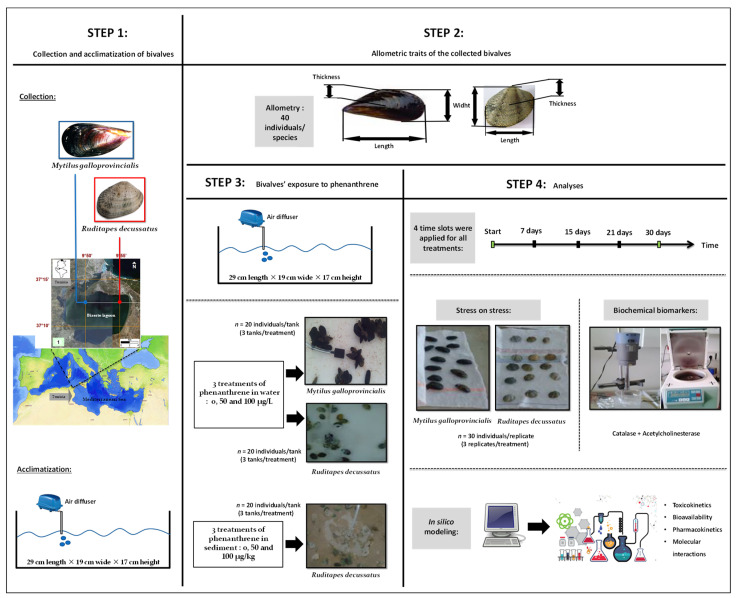
Graphical summary of steps and methodology adopted.

A graphical representation that shows the timeline and design of the experiment is given in Figure 1. Details of all schematic steps are presented below.

### 2.1. Bivalve Sampling and Experimental Set-Up

The bivalves *M. galloprovincialis* and *R. decussatus* were collected on the April 5, 2015 from Bizerte Lagoon (Tunisia), next to the Cities of Menzel Abderrahmane (37°13′43.7″ N, 9°51′45.7″ E) and Menzel Jemil (37°13′31.6″ N 9°55′40.9″ E), respectively. The bivalves were transferred to a cooling box containing lagoon water and transported to the laboratory (Figure 1). Afterwards, individuals were chosen randomly, regardless of sex (male or female) and size (*M. galloprovincialis*: 4.9 ± 0.5 cm, *R. decussatus*: 2.9 ± 0.2 cm) and placed in bins filled with water from their native habitat.

The bivalves were acclimatized for three days in tanks (29 cm length × 19 cm wide × 17 cm height) in an air-conditioned room. The water salinity, temperature, dissolved oxygen, and pH were maintained close to the values recorded in their natural habitat and measured on a daily basis with a thermo-salinity meter (LF 196; WTW, Weilheim, Germany), an oxymeter (OXI330/SET, WTW, Weilheim, Germany), and a pH meter (pH 330/SET-1, WTW, Weilheim, Germany), respectively. The tanks were constantly ventured with air-diffusers, allowing an even distribution of oxygen. A light/dark cycle was set at 12 h/12 h and the seawater was renewed every 24 h throughout the experiment. The temperature was kept at 18.03 ± 0.71 °C, the salinity at 37.16 ± 0.25 PSU, the pH at 8.2 ± 0.22, and the dissolved oxygen at saturation (9.54 ± 0.89 mg/L).

### 2.2. Contamination

Following the acclimatization period, a trial experiment was performed to better understand the response of bivalves to phenanthrene as a function of time and concentration. For each type of treatment, three tanks were considered, each comprising with 20 individuals. Stock solutions were prepared by dissolving phenanthrene (CAS Number 85-01-8 (Sigma-Aldrich, Co, St. Louis, MO, USA) in dimethyl sulfoxide (DMSO) (CAS Number 67-68-5, Sigma-Aldrich, Co, St. Louis, MO, USA). This procedure was performed before by dilution in lagoonal water, since the DMSO has no discernible effect on biomarkers [26]. The concentration-effect responses of mussels and clams were assessed by using two dosages of phenanthrene in water: 50 μg/L (hereafter WC1) and 100 μg/L (hereafter WC2) and paralleled by an Untreated Control where only the DMSO was involved (WUt). For clams only, a second experiment was set, with Untreated controls (SUt) and two sediment treatments: 50 µg/kg (hereafter SC1) and 100 µg/kg (hereafter SC2). The time-effect response was observed during four time slots (i.e., 7, 15, 21, and 28 days). The highest concentration in water (i.e., 100 μg/L) was chosen as such as to be approximately two thirds of the Lethal Concentration 50 (LC50) established for the mussel *M. edulis*, which is 148 µg/L [27]. The low concentration (i.e., 50 μg/L) of phenanthrene corresponded to approximately a third of the LC50 (50 µg/L). The phenanthrene concentrations used to contaminate sediments (i.e., 50 and 100 µg/kg) were within the range of Threshold Effect Levels (TEL) reported by the Canadian Council of Ministers of the Environment (CCME) [28] and MacDonald et al. [29] equal to 41.9 and 86.7 µg/kg, respectively.

### 2.3. Biometric Study

The mass and other allometric relations were measured for 40 individuals comprising mussels and clams. For each individual, the following parameters were measured:

- Length (L): the longest dimension from the dorsal to the ventral edge;
- Width (W): the longest dimension from the anterior to the posterior edge;
- Thickness (T): the longest dimension given by the convexity of both valves when gathered;
- Total Fresh Mass (TFM): the mass of a living individual with the shell cleaned of mud and water;
- Fresh Chair Mass (FCM): the mass of drained fresh visceral mass on filter paper;
- The Fresh Shell Mass (FSM): the mass of fresh wiped shell.

### 2.4. Stress on Stress

The *Stress on stress* concept was first described by [30] and is based on the rationale of measuring any given endpoint of molluscs exposed to two different types of stressors. As such, the bivalves were first exposed to anoxic conditions by placing them out of water. The molluscs are usually tolerant organisms to desiccation; however, their resistance to such stress is reduced if they were exposed already to desiccation in their natural habitats. Given this double type of disturbance, the most sensitive bivalves will die, whereas their strongest congeners will endure the treatments for longer periods of time. The *stress on stress* method is very simple and requires only a thermostatic room to store the animals in open air. By employing this method, the Median Lethal Time or the time until death of 50% of individuals (hereafter LT50) following exposure of a given population to a toxic substance or stressful condition can be assessed [31,32].

At the beginning of the *stress on stress* study, the mussels were divided into 36 groups, each comprising 30 individuals: 4 time slots (i.e., 7, 15, 21, and 30 days) × 3 treatments (i.e., control and two phenanthrene concentrations) × 3 replicates. For clams, the number of groups was doubled (72), due to the two different environments where the experiment took place: water and sediment. The bivalves were immediately exposed afterwards to anoxia in an air-conditioned room (18 °C, light-dark cycle 12 h/12 h) until death of all individuals was recorded. The dead specimens were removed every 24 h. We mention that the bivalves used in this experiment differed to those used to measure the biometry and in the evaluation of the enzymatic activities of biomarkers (Figure 1).

### 2.5. Enzymatic Biomarkers

#### 2.5.1. Sample Preparation for Total Protein Dosages

The control animals and those exposed to phenanthrene were opened by removing their valves, following the incision of the adductive muscles with a scalpel. After dissection, the animals were kept on ice at 4 °C to prevent protein denaturation and grounded afterwards in TBS buffer (Tris 50 mM, NaCl 150 mM) (pH 7.4) using a T25 Ultra-Turrax tissue homogenizer. The homogenate was centrifuged at 9000× *g* for 30 min at 4 °C. The supernatant (S9), comprising the post-mitochondrial fraction (i.e., cytosol, endoplasmic reticulum, and lysosomes), was stored in Eppendorf tubes at −80 °C until further biochemical analysis. The dosage of Total Protein was prepared according to the Bradford protocol [18]. The reagent Coomassy blue was used to interact with S9 proteins to produce a complex that absorbs light at 595 nm; the colour intensity is proportional to the amount of protein. The protein content was quantified using bovine serum albumin (BSA) as standard and equal loading was confirmed by electrophoresis by directly diluting the sample to a volume of 20 μL into sample buffer [33].

#### 2.5.2. Biochemical Analyses

The changes in optical density were quantified with a Beckman Du^®^ 520 type spectrophotometer. The activity of acetylcholinesterase was measured according to the colorimetric protocol described by [34]. This method evaluates the production rates of thiocholine from the acetylcholine by the cholinesterase into a buffer Na_2_PO_4_/NaHPO_4_ (0.05 M, pH 7.0) solution, 8 mM DTNB, and 45 mM acetylcholine. The released thiocholine reacts with 5-5-dithio-bis (2-nitrobenzoate (DTNB) to form 5-thio-bis-2-nitrobenzoate (TNB), a yellow product that absorbs light at 412 nm. The colour intensity is proportional to the amount of acetylcholinesterase in the sample. Following incubation of samples at 25 °C, the optical density was measured at 412 nm every 5 s for 30 min. Results are given as µmol/min/mg of hydrolysed substrate/total protein content, according to [35], which used this technique for microplate readers. The results are given as μmol/min/mg protein.

Catalase (hereafter CAT) is an enzyme that intervenes in the cell defence mechanisms against the oxidative stress. The activity of CAT (EC 1.11.1) was measured according to [36] and [37], with the aid of a Beckman Du^®^ 520 type spectrophotometer, bitec0, Minnesota, USA. The optical density was measured to assess the CAT activity at 240 nm, by the depletion rate of H_2_O_2_ in a reaction buffer solution Na_2_PO_4_/NaHPO_4_ (0.05 M, pH 7.0), 10 mM H_2_O_2_ [38]. The CAT activities are given as μmol/min/mg protein.

### 2.6. Dosage of Phenanthrene in Sediment and Water

Methods for extracting hydrocarbons from sediments were described by [39] and [40]. Soxhlet extraction with chloroform (1:2 *w*/*v*) for 8 h (40 °C) was performed on 50 g of dried sediment. The extract was then concentrated by rotary evaporation and separated into Non-Aromatic-(hereinafter referred to as NAH) and Total Aromatic (hereinafter referred to as TArom) hydrocarbons by liquid adsorption chromatography, using an alumina column and silica gel. The chemicals n-Hexane or n-Hexane/Chloroform (2:1) are considered solvents for NAH and TArom fractions, respectively. After solvent evaporation, TArom fractions containing all types of PAHs were analysed using a Hewlett-Packard 5890 gas chromatograph equipped with a temperature-controlled injector, flame ionization detector (GC/FID), and capillary column HP5: 5% diphenyl, 95% Dimethicone (25 m × 0.32 mm × 0.52 µm). For seawater, the separation and quantification of phenanthrene was performed by gas chromatography (GC) according to [41] and based on comparison with known standards injected under the same conditions using a certified standard reference (National Institute of Standards and Technology (NIST, Gaithersburg, MD, USA)). All analyses were performed in triplicate.

### 2.7. In Silico Analyses Analyses: Bioavailability, Toxicokinetics and Toxicodynamics

The toxicokinetics and inhibition of cytochrome P450 (CYP) isoforms of phenanthrene were assessed based on the ADME/Tox (for Absorption, distribution, metabolism, elimination and toxicity) attributes (i.e., absorption, distribution, metabolism, excretion, and toxicity) as previously described [42,43,44]. The bioavailability and toxicodynamics were also assessed based on the physico-chemical parameters [45,46,47].

### 2.8. Statistical Analyses

The biometric studies measure the relative growth of individuals. Hence, the following allometric equation was used: *Y* = *a X^b^*, where *b* is the allometric coefficient and *a* is the slope. After *log_e_*-transformations [48], the equation became *log_e_ Y* = *log_e_ a* + *b log_e_ X*, where *a* was the slope and *b* the intercept of the allometric regression. The biometric parameters were compared with Student *t*-tests. The intensity of the allometric growth was evaluated by comparing the values of *b* with the theoretical value of 1 for dimensions and with 3 for mass. If *b* = 1, the two parameters studied evolve in the same manner (i.e., isometric growth) with no significant differences (Student *t*-test). When *b* ≠ 1, the growth rate becomes allometric, with two potential scenarios: for *b* > 1, the growth rate of individuals is faster compared to that of reference populations (Student *t*-test: *p*-value < 0.05), whereas for *b* < 1, the growth rate is slower.

The changes in enzymatic activities were compared with one-way analysis of variance (1-ANOVA), followed by in-between multiple comparisons with Tukey’s HSD (Honestly Significant Difference) tests. To fulfil the normal distribution requirements for parametric tests, normality (Kolmogorov–Smirnov) and homogeneity of variances (Levene) tests were used. The statistical analyses were performed in STATISTICA v.8.

## 3. Results

### 3.1. Concentrations of Phenanthrene

The phenanthrene concentrations in the water and sediment collected on 5 April 2015 from Menzel Abderrahmane, in Bizerte Lagoon, Tunisia, were equal to 10.2 ± 0.14 µg/L and 8.8 ± 0.09 µg/kg, respectively. At the end of the bioassay, no significant changes in phenanthrene concentrations were registered with time (i.e., 7, 15, 21, and 28 days) for each type of treatment (df = 3, 1-ANOVA: *p*-value ˃ 0.05, Table 1). At each time slot, the phenanthrene concentrations in water or sediment showed a gradual significant increase as follows: Ut → C1 → C2 (df = 6, Tukey’s HSD comparisons: *p*-values < 0.0001, Table 1).

### 3.2. Biometric Study

All bivalves used in the current experiments for allometric measurements and mass were collected from the field and were not exposed to phenanthrene in the laboratory. Their allometric characterization was needed to assess a priori if they comprised morphometrically normal individuals compared to previous findings in Bizerte lagoon and for the appropriateness of the study.

#### 3.2.1. *Mytilus galloprovincialis*

**Figure 2:**
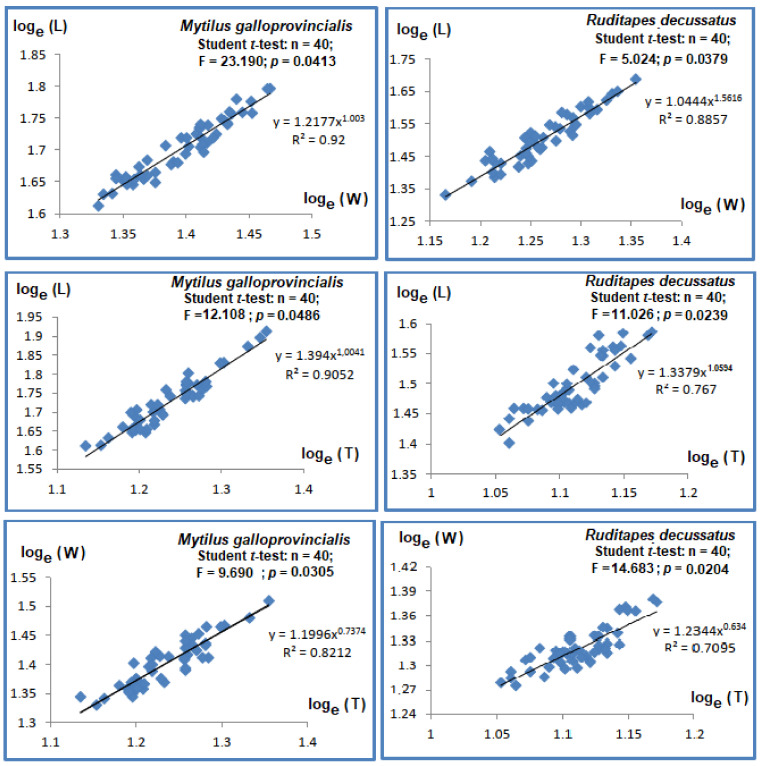
Regressions relating linear parameters in bivalve species *Mytilus galloprovincialis* and *Ruditapes decussatus*. Length (L); Width (W); Thickness (T).

The Pearson coefficients *R*^2^ between length, width, and thickness ranged between 0.821 and 0.92 and were significant (*p*-values < 0.001, see Figure 2). The regression slopes were significantly different to 1 (Student *t*-test ˃ 1.96, *p*-values < 0.05). The regressions between total length, width, and thickness (Figure 2) showed major allometry, whereas the regressions between width and thickness (Figure 2) showed minor allometry.

**Figure 3:**
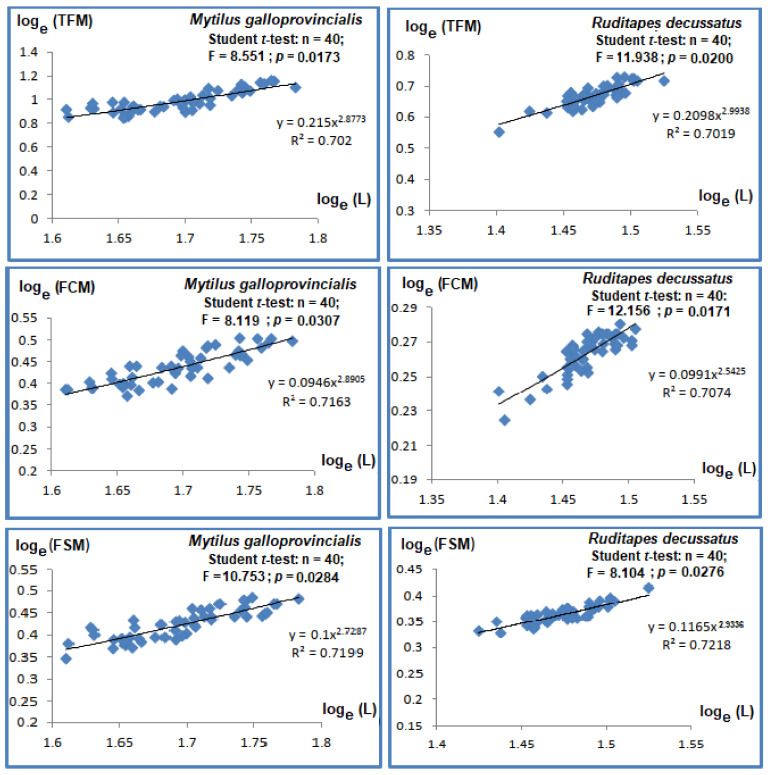
Regressions relating length and masses in bivalve species *Mytilus galloprovincialis* and *Ruditapes decussatus*. Total Fresh Mass (TFM); Fresh Chair Mass (FCM); Fresh Shell Mass (FSM), Length (L).

The relative mass growth rates were assessed through regressions between Total Fresh Mass (TFM), Fresh Chair Mass (FCM), Fresh Shell Mass (FSM), and length (L) (see Figure 3). The regressions among various types of masses to length were inter-correlated (*p*-values < 0.001, *R*^2^ > 0.7). The comparisons of slopes with Student *t*-tests to the theorical value of 3 supported significant differences between masses and length. The allometry was minor, given all slopes had values below 3.

#### 3.2.2. *Ruditapes decussatus*

The Pearson correlations between length and width were strong and significant (*R*^2^ = 0.767, *p*-value < 0.05), as with the correlations between length and thickness (*R*^2^ = 0.767, *p*-value < 0.05), with slopes significantly different and higher than unity (*p*-values < 0.05), showing major allometry (Figure 2). Student *t*-test showed significant differences (*p*-values < 0.05) between width and thickness, with minor allometry (slope = 0.634, Figure 2).

The Pearson coefficients of regressions between length and different types of masses varied between 0.701 and 0.721 and were significant (*p*-values < 0.05, Figure 3). The slope comparisons via Student *t*-test with the theoretical value of 3 led to the conclusion that the allometries were minor among the types of measured masses (i.e., TFM, FCM, and FSM).

### 3.3. Stress on Stress

#### 3.3.1. *Mytilus galloprovincialis*

**Figure 4:**
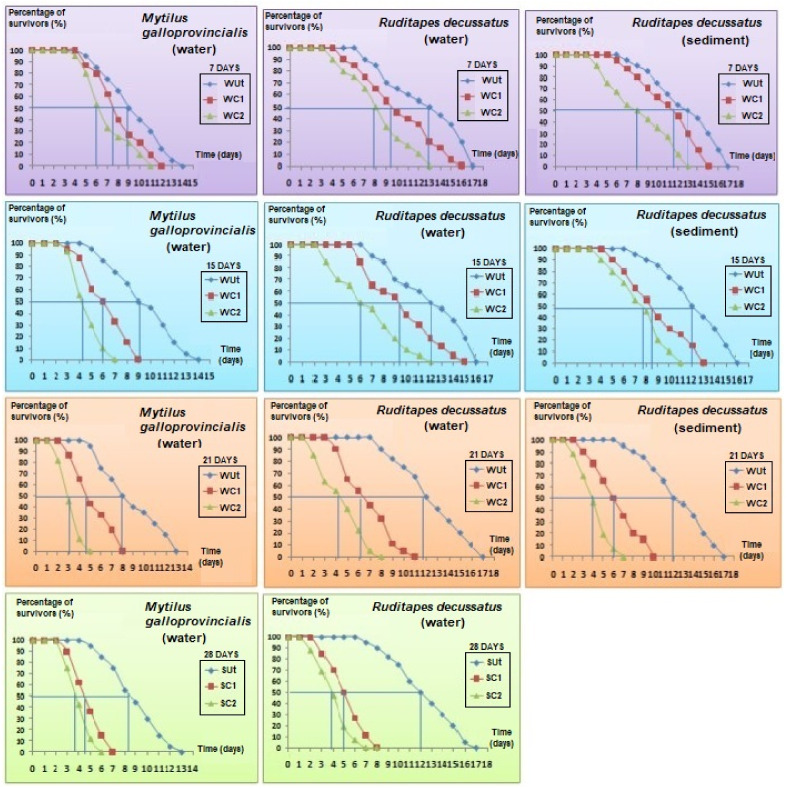
Percentage of survivors in bivalves’ species *Mytilus galloprovincialis* and *Ruditapes decussatus* in open air after being exposed to an Untreated control (Ut) and two concentrations of phenanthrene in water [50 µg/L (WC1) and 100 µg/L (WC2)] and sediment [50 µg/kg (SC1) and 100 µg/kg (SC2)] over 28 days.

The average LT50s ranged between 9 ± 0.21 to 8.15 ± 0.73 days in controls and were higher compared to treatments; the lowest LT50 were measured in WC2 for all time slots considered (1-ANOVA: df = 6, *p* < 0.01, Tukey’s HSD test: *p*-values < 0.01) (Figure 4). During the first 7 days of the experiment, LT50 decreased with 26%, from 8.6 ± 0.69 days for control to 6.4 ± 0.37 days when exposed to 100 µg phenanthrene/L (Figure 4). LT50 of WC2 was lower compared to WC1 for all time slots (Figure 4).

#### 3.3.2. *Ruditapes decussatus*

The LT50 of control clams reared in water did not change in time (1-ANOVA: df = 8, *p* = 0.892) (Figure 4). Thus, LT50 for control bivalves varied insignificantly between 13 ± 0.74 days at the beginning of the experiment to 11.7 ± 0.38 days after 28 days of exposure, with variation not exceeding 10%. On the other hand, after one week, LT50 values decreased by 22% in WC1 (1-ANOVA: df = 6, *p* = 0.025, Tukey’s HSD test: *p*-values < 0.01) and 37% in WC2 (1-ANOVA: df = 6, *p* < 0.01, Tukey’s HSD test: *p*-values < 0.001) compared to controls.

An additional experiment was performed with sediment that was either contaminated or not contaminated with phenanthrene. Given that 100% mortality rate was reached after 28 days of exposure, the results were only considered for 3 weeks (Figure 4). LT50 of control clams varied little, between 13 ± 0.24 to 11.93 ± 0.79 days (1-ANOVA: df = 8, *p* = 0.766) (Figure 4). After 7 days of exposure, LT50 decreased with 14% in SC1 (1-ANOVA: df = 6, *p* < 0.001, Tukey’s HSD test: *p*-values < 0.001) to 37% in SC2 (1-ANOVA: df = 6, *p* < 0.001, Tukey’s HSD test: *p*-values < 0.001), respectively, compared to control. This trend was statistically maintained in treatments compared to control during all time slots considered (Figure 4).

### 3.4. Biochemical Biomarkers

#### 3.4.1. *Mytilus galloprovincialis*

The AChE (Acetylcholinesterase) activity in the control was stable throughout the experiment (1-ANOVA: df = 4, F = 28.491, *p* = 0.844). In turn, the mussels treated with phenantrene recorded significant inhibitions of their enzymatic activity in time (1-ANOVA: df = 4, F = 50.110, *p* = 0.027, Tukey’s HSD test: *p*-values < 0.01). AChE inhibition was similar after 15 and 21 days, respectively, but dropped after 28 days of exposure.

**Figure 5:**
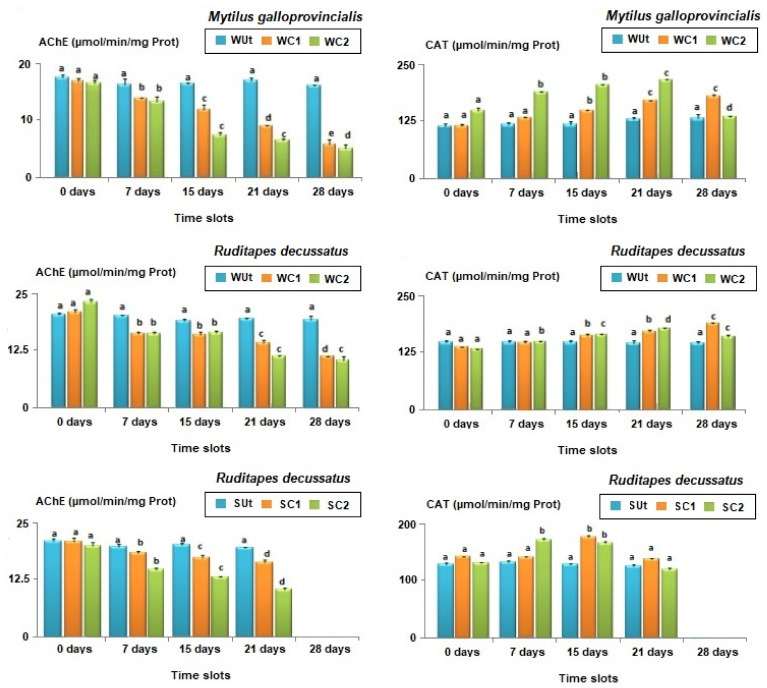
Average biomarker responses (± standard deviation) in bivalves’ species *Mytilus galloprovincialis* and *Ruditapes decussatus* after exposure to an Untreated control (Ut) and two concentrations of phenanthrene in water [50 µg/L (WC1) and 100 µg/L (WC2)] and sediment [50 µg/kg (SC1) and 100 µg/kg (SC2)] over 28 days. Catalase (CAT); Acetylcholinesterase (AChE). Different letters (a, b, c, d, and e) above bars indicate significantly differences from controls represented by ‘a’ after mutiple comparisions using Tukey’s HSD test.

The changes in CAT activity were measured at the same time intervals as for AChE (Figure 5). No significant changes were registered for CAT activities in the controls for all time slots considered (1-ANOVA: df = 4, F = 42.863, *p*-value = 0.857). In WC1 treatment, there were observed significant changes in the CAT activity after the 15th day of exposure (1-ANOVA: df = 4, F = 51.026, *p* = 0.045, Tukey’s HSD test: *p*-values < 0.01). In treatment WC2, there were significant increases in CAT activity after the 7th day of the experiment compared to controls observed, but they significantly dropped in the 28th day (1-ANOVA: df = 4, F = 104.008, *p* = 0.0314, Tukey’s HSD test: *p*-values < 0.001).

#### 3.4.2. *Ruditapes decussatus*

The concentrations of AChE were similar for control individuals during all time slots (i.e., 7, 15, 21, and 28 days, 1-ANOVA: df = 10, F = 44.109, *p*-value = 0.893, Figure 5). The AChE activities in WC1 significantly decreased (1-ANOVA: df = 10, F = 87.520, *p* = 0.037, Tukey’s HSD test: *p*-values < 0.05) after the 7th day of exposure, being reduced by 21.3%; the lowest levels were reached at the end of the experiment, with a 46.2% reduction (Figure 5). The AChE activities were also significantly lower in WC2 compared to WUt for the same time slot (7 days: 1-ANOVA: df = 6, F = 116.008, *p* = 0.0389/15 days: 1-ANOVA: df = 6, F = 92.145, *p* < 0.0257/21 days: 1-ANOVA: df = 6, F = 138.211, *p* < 0.01/28 days: 1-ANOVA: df = 6, F = 77.946, *p* < 0.01) (Tukey’s HSD test, *p*-values < 0.01, Figure 5).

No significant temporal variations were observed for AChE activity in control clams reared in sediment (1-ANOVA: df = 10, F = 107.241, *p*-value = 0.911, Figure 5). In contrast, the AChE activity in SC1 (7 days: 1-ANOVA: df = 6, F = 81.229, *p* = 0.0425/15 days: 1-ANOVA: df = 6, F = 135.104, *p* = 0.0146/21 days: 1-ANOVA: df = 6, F = 64.125, *p* < 0.01) and SC2 (7 days: 1-ANOVA: df = 6, F = 103.150, *p* = 0.0351/15 days: 1-ANOVA: df = 6, F = 78.134, *p* = 0.0221/21 days: 1-ANOVA: df = 6, F = 115.457, *p* < 0.01) significantly decreased compared to controls for all the time slots (Tukey’s HSD test, *p*-values < 0.01, Figure 5). Moreover, significant differences were also observed when the magnitude of AChE activity inhibition was compared in SC1 and SC2 (Student *t*-test: df = 4, F = 138.211, *p*-value = 0.0275).

The CAT activity in control clams was similar throughout the experiment (1-ANOVA: df = 6, F = 35.291, *p*-value = 0.816). By changing the exposure time for WC1 (1-ANOVA: df = 10, F = 218.443, *p*-value = 0.0127), significant changes in CAT activities were noticed. Thus, the bivalves from WC1 had first similar CAT levels with those from control following one week of exposure (Tukey’s HSD test: df = 4, *p*-value = 0.769). However, after the 15th day, significant differences were registered in CAT activities under WC1 compared to control (Tukey’s HSD test: df = 4, *p*-value = 0.0310, Figure 5). In WC2, the CAT reduction was significant compared to WC1 starting from the 7th day of exposure and continued until the 21st day (Student *t*-test: df = 4, *p*-values < 0.01, Figure 5). By the end of the experiment (i.e., 28 days) the activity of CAT under WC2 was significantly lower compared to WC1 (Student *t*-test: df = 4, *p*-values < 0.001, Figure 5).

Following the in vivo exposure of *R. decussatus* to phenanthrene in sediment, the CAT activity showed similar levels in control individuals no matter the exposure duration (1-ANOVA: df = 8, F = 64.527, *p*-value = 0.928). After 7 days of exposure (1-ANOVA: df = 6, F = 35.1291, *p* < 0.01), no significant effect was observed for SC1 (Tukey’s HSD: *p*-value = 0.928, Figure 5) but the increase was significant in SC2 for the same time slot (Tukey’s HSD: *p*-value < 0.001, Figure 5). After the 15th day, the CAT induction was significant (Tukey’s HSD test, *p*-values < 0.001 Figure 5) but thereafter, CAT activities dropped surprisingly compared to those found before at 7 and 15 days and no significant differences were found after 21st days of exposure compared to controls (1-ANOVA: df = 6, F = 201.846, *p*-value = 0.769).

### 3.5. In Silico Findings: Bioavailability and Toxicokinetics

**Figure 6:**
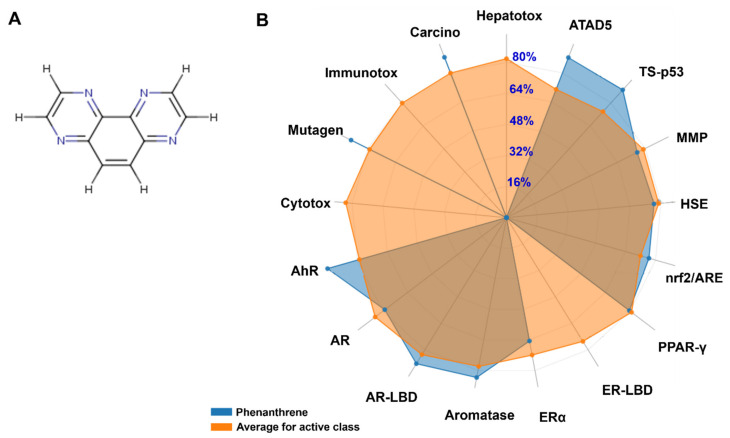
The chemical structure of phananthrene (**A**) and the average biomarker responses related to its toxicodynamics as compared to the average of class compounds (**B**). Cytox: Cytotoxicity; Mutagen: Mutagenicity; Immunotox: Immunotoxicity; Carcino: Carcinogenicity; Hepatotox: Hepatotoxicity; ATAD5: ATPase family AAA domain-containing protein 5; TS-p53: Tumor Supressor (Phosphoprotein) p53; MMP: Mitochondrial Membrane Potential; HSE: Heat shock factor response element; Nrf2/ARE: Nuclear factor like 2/antioxidant; PPAR-γ: Peroxisome Ploriferator Activated Receptor Gamma; ER-LBD: Estrogen Receptor Ligand Binding Domain; ERα: Estrogen Receptor Alpha; AR-LBD: Androgen Receptor Ligand Binding Domain; AR: Androgen Receptor; AhR: Aryl hydrocarbon Receptor.

The lipophilicity, bioavailability, and toxicokinetics data are given in Table 2. Both lipophilicity and bioavailability scores (0.55) confirm that phenanthrene is toxicologically active. The data are supported by the bioavailability radar (Figure 6). It was showed that phenanthrene is highly absorbable by the gastro-intestinal (GI) tract. It is also blood-brain barrier (BBB) permeable, but presented low skin permeability, as assessed using the Log Kp parameter. The possible inhibition of the major cytochrome P450 (CYP) isoforms was assessed. While phenanthrene was predicted to not inhibit cytochrome P450 (CYP) variants: CYP2C19, CYP2C9, CYP2D6, it instead inhibited CYP1A2 and CYP3A4, as well as the cytochrome P450 (CYP) variant, the CYP1A2. Both Tox21 stress response pathways (nrf2/ARE, HSE, MMP, TS-p53 and ATAD5) and Tox21 nuclear receptor signalling pathways (AhR, AR, AR-LBD, Aromatase, ER, ER-LBD and PPAR-γ) were inhibited following exposure to phananthrene. As showed in Figure 6, it is reported that these parameters presented significantly higher percentages, except for ER-LBD. Its toxico-dynamics revealed specifically, mutagenicity and carcinogenicity as endpoints.

## 4. Discussion

### 4.1. Does the Addition of Contaminated Water and Sediment with Phenanthrene Modify the Quality of the Environment in Treated Experimental Units?

The phenanthrene concentrations measured at the end of the bioassay in water and sediment were close to the targeted concentrations (see Table 1). These results also support the appropriateness of the adopted experimental conditions (i.e., 18 °C, light-dark cycle 12 h/12 h) that did not influence the reactivity of phenanthrene in water and sediment. Previously, ref. [49] focused on PAHs vaporisation from sediment (up to 60 °C) and observed that 10–30% of phenanthrene was detected in vapours after 100 days. We can conclude that under the current laboratory conditions, the vaporization rate of phenanthrene is negligible, at least for sediment (~5–10%).

At the end of the experiment, the treated microcosms contained significantly higher concentrations of phenanthrene in both water and sediment compared to the control, leading to the conclusion that the observed changes for *M. galloprovincialis* and *R. decussatus* were mainly induced by phenanthrene toxicity.

### 4.2. What the Biometric Features of M. galloprovincialis and R. decussatus Influenced by Exposure to Phenanthrene?

The characterization of biometric traits is important since the bivalves were collected from the Bizerte lagoon, which is normally exposed to several stressors [50]. Therefore, in the first instance, the realistic estimation of exposed bivalves as being appropriate for the laboratory experiment was a crucial step. It is known that the morphology of bivalves is affected by stressors [51,52]. The linear (i.e., length, width, and thickness) and mass parameters (i.e., Total Fresh Mass, Fresh Chair Mass, and Fresh Shell Mass) were measured in the current experiment to assess if the phenantrene influenced the growth rate of bivalves. The growth rates for length and width were faster compared to thickness for the mussel *M. galloprovincialis*, but the growth rate of mass was lower compared to that of length. For the clam *R. decussatus*, the increase rate of length was the highest, for thickness intermediary, and the lowest for width. This trend may explain the triangular shape and the relative thickening of the valves, allowing the stability of individuals in sediments due their benthic lifestyle. The growth rate of the total mass was also lower compared to length. This is probably due to the fact that the tested clams were in the post-laying phase, following the release of the gonadic content. According to [53,54,55], the compression of the shell in mussels is strongly influenced by two additional factors: the relative growth rate of linear variables and the density of individuals. Thus, a lower compression of the shell is related to slower growth rates and population density. Ref. [56] showed for the bivalves *M. galloprovincialis* and *R. decussatus,* an intimate relation among linear parameters, such as lengths, width, and thickness exist, and that the relative growth of mass was proportional to that of length. The growth rate of bivalves is equally influenced by water physic-chemistry, such as temperature, pH, photoperiod, emersion time, and air temperature during tides [56,57], but also by the availability of food [58,59]. All these features seem to indicate that the collected individuals from Bizerte lagoon (Tunisia) were normal, since no significant biometric anomalies were registered compared to the common shapes of both species considered.

### 4.3. Does the Phenanthrene Influence the Responses of M. galloprovincialis and R. decussatus to Stress on Stress?

Ideally, laboratory techniques and biomonitoring tools should be accurate, reliable, and cheap. In order to overcome the drawbacks imposed by reality, the *stress on stress* approach was implemented [30].

In the current study, the comparison of the tolerance to anoxia of *R. decussatus* compared to *M. galloprovincialis* showed that under the same environmental conditions, the former clam was more tolerant to anoxia compared to the latter mussel. This could be explained by different metabolic pathways and defence mechanisms of the two bivalves [38]. The shell closure of clams was tighter compared to that of mussels; the latter group needs the aperture for the protrusion of byssus, allowing the entrance of toxic substances at higher rates compared to clams [56].

The relative constancy of LT50 throughout the experimental may indicate a stability of the experimental conditions and that control bivalves better tolerated the open-air conditions compared to their congeners from contaminated treatments. However, the comparison of our results with those from literature (Table 3) showed that the tolerances were higher compared to those of bivalves collected from the same site of Menzel Abderrahmane (Bizerte Lagoon, Tunisia) by [60], but similar to the values observed by [56]. The contamination with phenanthrene reduced the LT50 in a time- and concentration-dependent way, altering the physiological status of both bivalves. The time and concentration dependency comprise typical hallmarks of expectable dose-response relationships: the toxicity typically increases with higher concentrations and with longer exposure durations, as previously demonstrated by [61]. Previous studies support the potential direct toxic effect of phenanthrene for bivalves [62,63,64]. PAHs are known for their carcinogenic, teratogenic, and mutagenic properties for aquatic organisms and humans [65]. Most PAHs have strong hydrophobic characteristics and tend to be absorbed by organic and inert suspended particles in water and finally accumulate in sediments, mainly applicable for the heaviest compounds [66,67,68,69,70]. Therefore, organisms with sessile lifestyles, such as *M. galloprovincialis* and with benthic preferences such as the European clam *R. decussatus,* could be doubly exposed to these contaminants, namely in the water column and at the water–sediment interface [21].

The values of LT50 registered for *R. decussatus* support the assertion of higher toxicity of phenanthrene following the sediment contamination compared to water exposure, given that the LT50 declined faster in the former *milieu*. This result is in accordance with that of [71] in contaminated sediments with pesticides. The results of the current study showed that the sensitivity thresholds to phenanthrene for both bivalves decreased with exposure time.

### 4.4. Do Enzymatic Biomarkers Respond to Phenanthrene Contamination?

The concentrations of AChE and CAT were similar in time for control bivalves, suggesting that the experimental conditions had no significant effect on the activities of biomarkers for *M. galloprovincialis* and *R. decussatus*. Moreover, the specimens reared in the laboratory kept a healthy appearance and normal behaviour after 28 days. However, the inhibitory effect of phenanthrene was observed early in the experiment (i.e., after 7 days) and led to a maximum inhibition after 28 days. These results highlight the detrimental effects following exposure to this PAH, probably through bioconcentration in the tissues of bivalves and potential neurotoxic actions. These detrimental effects were observed for the mussel *M. galloprovincialis* in water and the clam *R. decussatus* in water and sediment contaminated with phenanthrene. The detrimental effects of phenanthrene on AChE activity became more visible for clams compared to mussels after 7 days of exposure. However, it can overall be concluded that the effects of contamination with phenanthrene were similar for both tested species. Comparable results were reported by [72,73], who showed the persistent inhibition of the AChE activity in the digestive glands of *M. galloprovincialis* after two days of exposure to benzopyrene. These authors reported that the reduction of the AChE activity was the result of an overall collapse of the health status of the animals following exposure to benzopyrene. Moreover, ref. [74] suggested that strong inhibitions of AChE activities may lead to the tetanization of muscles, and finally to the death of bivalves. Ref. [75] found similar impacts on gills and digestive glands following exposure of *M. galloprovincialis* and *R. decussatus* to benzo[a]pyrene. These findings, corroborated with similar effects following the exposure to organophosphorus and carbamates pesticides, which are considered as one of the most neurotoxic compounds, support the strong inhibition of AChE activity of bivalves by phenanthrene [75,76,77,78,79].

For the clam *R. decussatus* exposed to phenanthrene in water and sediment, the inhibition of AChE indicates a double effect. This may be achieved through the adsorption capacity of phenanthrene in sediment particles, which increases its bioavailability for benthic bivalves. Our results showed that the response of the antioxidant activity was similar in *R. decussatus* and *M. galloprovincialis* and that significant induction was observed for the former bivalve following contamination of water, as well as for the latter species in water and sediment. Following the exposure in the water column, the response was clearer for clams compared to mussels and started earlier for the highest concentration (i.e., 100 μg/L). A comparable trend was also registered for clams reared in contaminated sediments. Similar cases of early induction on the catalase activity were observed for *Perna viridis* exposed to PAHs [80] and *Bathylomodiolus azoricus* exposed to metals (i.e., mercury and copper, see [81]). Our results are convergent with those reported by Bebianno and Barreira (2009), who proved the strong accumulation of the most soluble PAHs in *R. decussatus* after just one day of exposure. The exposure to PAHs led to increased catalase activity in the mussels *M. edulis* [82], *Perna viridis* [20], *Mya arenaria*, and *M. trossulus* [83]. Moreover, ref. [84] showed that anthracene, benzopyrene, and other petrochemicals enhance the catalase activity in the fish *Pomatoschistus microps*. The enzyme catalase is normally present in many types of tissues and organs, intervening in cell defence mechanisms against oxidative stress by eliminating Reactive Oxygen Species (ROS) and accelerating the spontaneous reaction of dismutation of hydrogen peroxide (H_2_O_2_), which is toxic [85]. This enzyme prevents the peroxidation of biomolecules induced by H_2_O_2_. The catalase is sensitive to xenobiotics, such as PAHs, Polychlorinated biphenyls (PCBs), metals, and pesticides, known to induce oxidative stress in cell membranes and damages by protein oxidations, lipid peroxidations, and the formation of DNA adducts [80,81,86,87,88]. The low induction of catalase noticed at phenanthrene concentrations smaller than 50 µg/L could be explained by the fact that the oxidative stress, following the contamination, was first neutralized by antioxidant mechanisms rather than the enzymatic pathways. In the first step following exposure to toxic substances, the tissues trap ROS through numerous non-enzymatic compounds, such as vitamins (i.e., A, C and E), ubiquinone, carotenoids, flavonoids, and uric acid. Once the free ROS exceeds the capacity of these trappers, the antioxidant enzymes such as the catalase are mobilized, potentially explaining their concentration in bivalves until the 21st day of the experiment.

A delayed response was found for mussels and clams in treatments contaminated with the highest concentrations of phenanthrene after 28 days of experiment, given the significant collapse registered in the CAT activity. Ref. [85] reported an initial phase of increasing CAT activity in the digestive glands of the clam *R. decussatus* beginning after three days and reaching a maximum after 28 days of exposure, but followed by a decrease afterwards.

The contamination with phenanthrene may have also induced some behavioural changes for the reared on treated substrate *R. decussatus*. These clams were unable to close hermetically their valves, leaving the siphons extended outwards. These results are similar with previous works that reported changes in the behaviour of the bivalves *Macoma balthica*, *Corbicula flumea* and *Crassostrea gigas,* such as modifications in valves’ movement and reduced filtration rate following exposure to organic contaminants [89].

Regarding the in silico analyses, our results confirmed the possible toxicological outcomes of exposure to phenanthrene. It has been shown that phenanthrene is highly absorbed by the GI tract. It is also BBB permeable, but presented low skin permeability as assessed using Log Kp parameter. The possible inhibition of the major cytochrome P450 (CYP) isoforms was assessed. The phenanthrene inhibited both CYP1A2 and CYP3A4 but not CYPC19, CYPC9, and CYP2D6. These results confirmed recent results by Hedfi et al. (2022) [90] regarding phenanthrene and chrysene intoxication in nematodes. Tox21 stress response pathways (nrf2/ARE, HSE, MMP, TS-p53, and ATAD5) and Tox21 nuclear receptor signalling pathways (AhR, AR, AR-LBD, Aromatase, ER, ER-LBD, and PPAR-γ) are commonly assessed in toxicological approaches [90]. Excepting ER-LBD, all assessed pathways presented significant, high percentages, with associated mutagenicity and carcinogenicity, similar to those observed following exposure to polycylic aromatic hydrocarbons [90,91,92].

## 5. Conclusions

Following exposure to phenanthrene, the LT50 of bivalves decreased for the used concentrations and exposure time, a direct effect of the detrimental actions of this PAH on the physiological status of mussels and clams. The detrimental effects of phenanthrene were also reflected in a time- and concentration-dependent manner, with better tolerance observed for the clam *R. decussatus* compared to the mussel *M. galloprovincialis*. In the case of biochemical biomarkers, the effects were also time- and concentration- dependent. The AChE activity was inhibited in the bivalves after contamination with phenanthrene of water (i.e., the mussels and the clams) or just sediment (i.e., clams). In turn, the CAT activity was induced at a faster rate in mussels compared to clams until the 21st day of the experiment, afterwards followed by a collapse of the physiological status, reflected in the decrease of the enzyme activity in the 28th day of the experiment. The in silico findings (i.e., bioavailability, toxicokinetics, and toxicodynamics) supported and explained the measured morphological and physiological results, based on the biometric, *stress on stress*, and analyses of biomarkers.

## Figures and Tables

**Table 1:** Targeted and actual concentrations of phenanthrene measured in water and sediment where two bivalve species (*Mytilus galloprovincialis* and *Ruditapes decussatus*) were reared and exposed to an Untreated control (Ut) and two concentrations of phenanthrene in water [50 µg/L (WC1) and 100 µg/L (WC2)] and sediment [50 µg/kg (SC1) and 100 µg/kg (SC2)] for 28 days. Different letters (a, b, c, A, B, and C) indicate significant differences with the correspondent untreated controls, represented by ‘a’ for *Mytilus galloprovincialis* and ‘A’ for *Ruditapes decussatus* (*n* = 3, *log*-transformed data, Tukey’s HSD test: *p* < 0.05).

Targeted Concentration	Species	7 Days	15 Days	21 Days	28 Days
WUt (0 µg/L)	*Mytilus galloprovincialis*	10.2 ± 0.19 **(a)**	10.0 ± 0.08 **(a)**	9.9 ± 0.03 **(a)**	10.0 ± 0.06 **(a)**
	*Ruditapes decussatus*	9.8 ± 0.19 **(a)**	10.1 ± 0.07 **(a)**	9.6 ± 0.10 **(a)**	10.3 ± 0.13 **(a)**
WC1 (50 µg/L)	*Mytilus galloprovincialis*	45.2 ± 2.11 **(b)**	47.1 ± 5.61 **(b)**	42.6 ± 4.08 **(b)**	44.5 ± 7.28 **(b)**
	*Ruditapes decussatus*	44.9 ± 1.07 **(b)**	47.4 ± 5.52 **(b)**	43.1 ± 6.08 **(b)**	44.8 ± 3.20 **(b)**
WC2 (100 µg/L)	*Mytilus galloprovincialis*	92.7 ± 2.85 **(c)**	92.3 ± 6.10 **(c)**	84.1 ± 4.68 **(c)**	87.9 ± 8.04 **(c)**
	*Ruditapes decussatus*	92.4 ± 3.07 **(c)**	90.9 ± 5.46 **(c)**	86 ± 5.32 **(c)**	88.2 ± 6.27 **(c)**
SUt (0 µg/kg)	*Ruditapes decussatus*	8.9 ± 1.09 **(A)**	8.5 ± 0.62 **(A)**	8.6 ± 1.10 **(A)**	8.6 ± 1.17 **(A)**
SC1 (50 µg/kg)	*Ruditapes decussatus*	46.1 ± 5.01 **(B)**	42.5 ± 2.18 **(B)**	44.8 ± 3.37 **(B)**	45.9 ± 4.04 **(B)**
SC2 (100 µg/kg)	*Ruditapes decussatus*	90.2 ± 5.12 **(C)**	92.3 ± 3.75 **(C)**	94.2 ± 5.06 **(C)**	90.1 ± 6.14 **(C)**

**Table 2:** Toxicokinetic properties of phenanthrene based on its physico-chemical and ADME/Tox (for absorption, distribution, metabolism, elimination, and toxicity) attributes.

Lipophilicity		Physicochemical Properties		
Log *P_o_*_/*w*_ (iLOGP)	Consensus Log *P_o_*_/*w*_	TPSA (A^2^)	Log S (Ali)	No. rotatable bonds
1.54	1.12	51.56	−1.14	0
**Toxicokinetics**				
GI absorption	BBB permeant	P-gp substrate	Log Kp (cm/s)	Bioavailability score
High	Yes	No	−7.06	0.55
CYP1A2 inhibitor	CYP2C19 inhibitor	CYP2C9 inhibitor	CYP2D6 inhibitor	CYP3A4 inhibitor
Yes	No	No	No	Yes

Log *P_o_*_/*w*_: Lipophicity; TPSA: Topological Polar Surface Area; Log S (Ali): Water Solubility; GI: gastro-intestinal; BBB: Blood-Brain Barrier; P-gp: P-Glycoprotein; CYP: Cytochrome P450.

**Table 3:** Comparison with data from literature on Median Lethal Time of 50% (LT50) of the populations of *Mytilus galloprovincialis* and *Ruditapes decussatus* after exposure to two concentrations of phenanthrene in water [50 µg/L (WC1) and 100 µg/L (WC2)] and sediment [50 µg/kg (SC1) and 100 µg/kg (SC2)] over 28 days.

Reference	Location	Species	LT50
Khessiba (1999)	Bizerte lagoon, Tunisia	*Ruditapes decussatus*	5.75
		*Mytilus galloprovincialis*	4.16–8.17
Dellali (2001)	Bizerte lagoon, Tunisia	*Ruditapes decussatus*	6–8.66
		*Mytilus galloprovincialis*	6.23–6.67
Current study	Bizerte lagoon, Tunisia	*Mytilus galloprovincialis* (WUt)	9
		*Ruditapes decussatus* (Wut)	13
		*Ruditapes decussatus* (SWt)	13
	Bizerte lagoon, Tunisia	*Mytilus galloprovincialis* (WC1)	4.31–7.42
		*Ruditapes decussatus* (WC1)	4.73–9.49
		*Ruditapes decussata* (SC1)	5.86–10.73
	Bizerte lagoon, Tunisia	*Mytilus galloprovincialis* (WC2)	2.77–6.4
		*Ruditapes decussatus* (WC2)	3.85–7.67
		*Ruditapes decussatus* (SC2)	3.73–7.82

## Data Availability

All the data in the article are available from the corresponding author upon reasonable request.
